# Europe Needs a Central, Transparent, and Evidence-Based Approval Process for Behavioural Prevention Interventions

**DOI:** 10.1371/journal.pmed.1001740

**Published:** 2014-10-07

**Authors:** Fabrizio Faggiano, Elias Allara, Fabrizia Giannotta, Roberta Molinar, Harry Sumnall, Reinout Wiers, Susan Michie, Linda Collins, Patricia Conrod

**Affiliations:** 1 Department of Translational Medicine, Università del Piemonte Orientale Amedeo Avogadro, Novara, Italy; 2 School of Public Health, University of Turin, Torino, Italy; 3 Child and Baby Lab, Department of Psychology, Uppsala University, Uppsala, Sweden; 4 Department of Public Health Sciences, Karolinska Institutet, Stockholm, Sweden; 5 Centre for Public Health, Liverpool John Moores University, Liverpool, United Kingdom; 6 Department of Psychology, University of Amsterdam, Amsterdam, Netherlands; 7 Department of Clinical, Educational and Health Psychology, University College London, London, United Kingdom; 8 The Methodology Center and Department of Human Development and Family Studies, Pennsylvania State University, State College, United States of America; 9 Addictions Department Institute of Psychiatry, King's College London, London, United Kingdom; 10 Department of Psychiatry, University of Montreal, Montreal, Québec, Canada

## Abstract

Fabrizio Faggiano and colleagues discuss how a central, transparent, and evidence-based approval process is needed for behavioral prevention interventions in Europe and propose a way forward.

*Please see later in the article for the Editors' Summary*

**Summary Points**

- Prevention interventions tackling health-compromising behaviours have the potential to play a major role in reducing the burden of noncommunicable diseases in Europe and other areas of the world. However, in Europe, no prior evaluation is required for the implementation of prevention interventions, thus leading to widespread dissemination of potentially ineffective or harmful interventions.
- A central, transparent, evidence-based, context-aware, and research-oriented approval process for behavioural prevention interventions is likely to foster the implementation and dissemination of effective interventions in Europe.
- Similarly to medicine approval systems, such a new approval process could be based on four consequential phases evaluating the effect of the following: single components (phase 1); combinations of components (phase 2); the final intervention—comprising only components found effective in the previous phases—via large, multicentre, randomized trials whenever possible (phase 3); and the long-term effects as well as the effects in different contexts (phase 4).
- Once phase 3 shows convincing results, the intervention would be approved for delivery to its target population.
- An approval process for behavioural prevention interventions is likely to lead to positive consequences both for practice, by strengthening the role and impact of prevention in times of limited economic resources, and for research, by promoting the robust evaluation of all promising prevention interventions.

## Introduction

Seven risk factors account for 56.1% of the attributable disability-adjusted life years (DALYs) in western Europe: dietary risks, smoking, high blood pressure, high body mass index (BMI), physical inactivity, excessive alcohol consumption, and high fasting plasma glucose [1]. Although such a figure reflects the predominant burden of noncommunicable diseases (NCDs) in high-income countries, it is becoming a priority also in middle-income and low-income countries [2]. By addressing such risk factors, prevention and health promotion can play a major role in reducing the burden of NCDs. The crucial function of prevention in tackling the NCDs epidemic is shared globally, as highlighted by the WHO programme “Gaining Health” and, more recently, by the United Nations High-Level Meeting on NCDs—in which prevention has been included among the five priority actions needed globally and nationally to respond to the NCDs epidemic [3],[4].

What distinguishes prevention of NCDs from the more traditional prevention activities of communicable diseases is the aim to avoid or change health-compromising behaviours or to promote healthy behaviours. Prevention of NCDs includes individual and environmental intervention, e.g., family-based interventions tackling alcohol misuse, national policies prohibiting indoor smoking, school-based education to foster correct eating behaviour, walking groups for children or adults, taxation of tobacco or alcohol products, and policies to limit junk food in vending machines on school premises. This aim is particularly critical and requires much more caution than traditional prevention practices, for example, those intended to control environmental pollution and infectious diseases.

In Europe, a number of official documents, both at the regional and country level, promote an evidence-based approach to prevention [3],[5],[6]. However, there is no regulatory system for the implementation of behavioural prevention interventions. This contrasts with the situation in clinical medicine, in which there is a long-standing culture of using robust evidence to inform commissioning and clinical decisions. The European Medicines Agency (EMA) and European national authorities manage a well-established, although not perfect [7], system for the assessment of safety and effectiveness of drugs.

With this contribution, we aim to initiate a debate about the need for a unique European evaluation and approval system of prevention interventions for health-compromising behaviours.

## Need for a Rigorous Evaluation of Behavioural Prevention Interventions

In Europe, interventions for preventing health-compromising behaviours can be implemented and disseminated without any preliminary authorisation, whatever setting (school, family, and community), professional, or type of method and technology involved. This is of concern for both ethical and economic reasons. First—and contrary to common belief—prevention interventions are not just harmless or ineffective in the worst-case scenario. They may also be harmful. Iatrogenic effects have been observed in interventions tackling risky behaviours such as physical inactivity, substance misuse, early sexual intercourse, and juvenile delinquency [8]–[12]. It is ethically unacceptable that a prevention intervention could significantly increase BMI, or tobacco or alcohol use, or frequency of cannabis use, or pregnancies and sexually transmitted diseases. Quoting the “father” of evidence-based medicine, David Sackett, “[…] the presumption that justifies the aggressive assertiveness with which we go after the unsuspecting healthy must be based on the highest level of randomized evidence that our preventive manoeuvre will, in fact, do more good than harm” [13].

Second, resources are likely to be wasted if evidence of effectiveness is missing or not sought. Cochrane reviews on the prevention of risky behaviours [14]–[16] show that effective interventions are in the minority of those evaluated by randomized studies. There is no reason to presume that non-evaluated interventions may be more effective than those that underwent rigorous evaluation. The resource allocation in the development and delivery of ineffective interventions is of particular concern in these times, given Europe's overstretched health systems.

## Need for Improving the Analysis and Description of Mechanisms of Behavioural Prevention Interventions

Prevention interventions for health-compromising behaviours usually target psychological, social, and organisational factors hypothesised to mediate the associations between intervention and behavioural outcomes. Although theories should play a crucial role in the design and evaluation of prevention interventions, there is a lack of awareness and consensus as to which theories should be applied and what method should be used [17],[18]. Many interventions are an amalgam of approaches and contents that do not explicitly draw on formal theories; others mention theory but are not truly theory-driven and do not always adhere consistently to a theory's tenets, being driven by implicit common sense models of behaviour [19],[20].

Moreover, interventions are usually delivered as a complex combination of components (“active ingredients” targeting different mediators), both in terms of contents, activities, techniques, and modes of delivery. However, the interventions are usually poorly described. Less than 30% of reports of randomized studies present a detailed description of the intervention allowing accurate replication and implementation, and fewer include descriptions of mechanisms of action [19],[21]. In addition, complex interventions, composed of several components, are usually evaluated together in randomized studies, which makes it difficult to disentangle the effect of a single ingredient on mediators and behavioural outcomes. The failure to conceptualize, define, and describe intervention components and mediators restricts the potential for evaluation to add evidence about effective interventions and mechanisms of action. In addition, the effects of context are rarely recognized, reported, or analysed.

Not knowing why, how, and where prevention interventions work limits knowledge about generalizability and optimization of interventions. It also increases the cost of implementation, as non-essential mediators might be inappropriately targeted and non-essential components may be inadvertently included.

If mediators of the target behaviour are identified, it is easier to design intervention components that are more likely to be effective. If the intervention components most strongly associated with effectiveness are known, more accessible, practical, and lower-cost, yet still effective, prevention interventions can be elaborated and disseminated. Moreover, they can be adapted to meet local needs and implemented in situations that are less ideal than research circumstances.

The complexity of behavioural prevention interventions, together with the lack of accurate reporting of mechanisms of action and analysis of the effects of components and their interactions, has serious consequences for prevention science: new interventions and evaluations occur in relative isolation, limiting the possibility of building an incremental technology of prevention [19].

## Identifying and Selecting Evidence-Based Behavioural Prevention Interventions: Current Situation

Prevention guidelines are uncommon and usually of mixed quality, and no national or international systems exist for the regulation of effective interventions. Prevention professionals usually have to search and appraise the literature by themselves if they want to select evidence-based interventions to transfer into practice.

There are some local experiences of public registries of evidence-based interventions in some areas of prevention [22]–[24]. However, at least two reasons suggest that these registries may not be enough to guide practice. First, they have a weak level of global authority; thus, they cannot limit the proliferation of unevaluated or harmful interventions. Secondly, they present a great variability in the level of evidence required to define an intervention as effective, and in the way they help dissemination. This is obviously a potential cause of uncertainty for decision-makers and implementers. Table 1 compares the criteria for intervention classification adopted by seven registries considered by Gandhi et al. [22], to which we added two European resources, the European Monitoring Centre for Drugs and Drug Abuse (EMCDDA) 's Best Practice Portal and the Dutch Recognition System [23],[24]. Although evidence of efficacy and quality of evaluation are considered by all registries, aspects such as quality of programme contents, programme implementation methods, and programme replicability are considered only in four out of nine registries. In this panorama the attempts to define standards of quality of prevention intervention, for example, from the Society for Prevention Research (see at www.preventionresearch.org) and from the EMCDDA (see at www.emcdda.europa.eu), do not appear to have had any visible effects.

**Table 1:** Criteria used in most widespread registries of evidence-based prevention interventions.

	Criteria							
List of registries	Evidence of efficacy	Quality of evaluation	Quality of programme goals	Quality of programme rationale	Quality of programme content and appropriateness	Quality of programme implementation methods	Educational significance^a^	Usefulness and replicability
Blueprints	Yes	Yes	Yes	N/A	N/A	N/A	N/A	Yes
Drug strategies: Making the Grade	Yes	Yes	Yes	N/A	N/A	N/A	N/A	N/A
ED List	Yes	Yes	Yes	Yes	Yes	Yes	Yes	Yes
Maryland Report	Yes	Yes	N/A	N/A	N/A	N/A	N/A	N/A
NIDA Guide	Yes	Yes	N/A	N/A	N/A	N/A	N/A	N/A
SAMHSA National Registry of Evidence-Based Programs and Practices	Yes	Yes	Yes	Yes	Yes	Yes	No	Yes
Youth Violence: A Report of the Surgeon General	Yes	Yes	Yes	N/A	N/A	N/A	N/A	Yes
EMCDDA Best Practice Portal	Yes	Yes	Yes	No	Yes	Yes	No	No
Dutch Recognition System	Yes	Yes	Yes	Yes	Yes	Yes	No	Yes

This table is partly based on the work by Gandhi et al. [22], with the addition of the EMCDDA Best Practice Portal [23] and the Dutch Recognition System [24].

a The application describes how the intervention is integrated into schools' educational mission.

Abbreviations: EMCDDA, European Monitoring Centre for Drugs and Drug Abuse; NIDA, National Institute on Drug Abuse; SAMHSA, Substance Abuse and Mental Health Services Administration; ED, US Department of Education; N/A, not available.

## Existing Frameworks for the Regulation of Interventions

Improving the regulation of prevention interventions for health-compromising behaviours to ensure that effective interventions are implemented and disseminated is likely to be challenging.

In clinical practice, authorization agencies such as the United States Food and Drug Administration (FDA) and the European Medicine Agency (EMA) are appointed to manage an evidence-based evaluation process intended to guarantee that only safe and effective drugs will be approved for marketing. Although formal pathways are slightly different, for both agencies the process is based on a four-step evaluation: small trials to test pharmacodynamics, pharmacokinetics, and dosage (phase 1); medium trials for assessing efficacy and short-term effects (phase 2); large, randomized trials to evaluate effectiveness and side effects (phase 3); and post-marketing surveillance and additional studies for specific subgroups of patients and assessment of rare side effects (phase 4) [25]. Pharmaceutical companies apply for EMA and/or FDA approval by transmitting all the preclinical and clinical information obtained during the first three phases [26]. Approval is a necessary prerequisite for marketing a drug in Europe and in the United States.

A systematic approach to developing and evaluating complex prevention interventions, as the majority of prevention interventions are, has been developed by the United Kingdom's Medical Research Council (MRC) [27],[28]. The first set of guidance proposed a process for the evaluation of complex interventions, which is logically consistent with the sequential phases of the drug approval process. The second set was based on a more sophisticated understanding of complexity and called for the defining of relevant intervention components, as well as underlying mechanisms and theories (modelling phase), testing acceptability and feasibility (pilot phase), evaluating effectiveness in an experimental study (evaluation phase), and assessing long-term effects in uncontrolled settings (implementation phase). The updated guidance in 2008 conceptualised the process as a cycle, emphasising the importance of considering implementation right at the beginning of the intervention development process.

Collins and colleagues addressed another issue that is not shared by drug registration systems: the complexity of interventions [29],[30]. In order to evaluate the role of each single component of prevention interventions, they suggest adopting a multiphase optimization strategy, which may involve the application of a factorial design in order to assess the independent role of each component (see at http://methodology.psu.edu/ra/most).

These approaches, no matter how innovative, however, are not applicable to policy evaluation, a strong component of preventive strategies. In order to keep the same level of validity of the assessment, this requires tailored approaches to evaluation [31], as those developed for tobacco control [32].

## A Proposal for a System of Evaluation and Approval of Behavioural Prevention Interventions

To tackle the overuse of interventions without scientific evidence and the underuse of effective interventions, Europe needs an approval system for prevention of health-compromising behaviours. This system would allow decision-makers and implementers to access the necessary information and materials to select the best prevention intervention for any specific need (e.g., target behaviour, population, setting, available resources, etc.). This system should be:

- Based on evidence. It should rely on the most valid evaluation approach for the specific intervention to assess. If randomized controlled trial would not be a feasible option, for example, for policy evaluation, the system should include alternative research designs that allow for relatively strong causal inferences (e.g., cohort design or interrupted time series design).
- Aware of context. Contextual moderators are of great importance for prevention of health-compromising behaviours and should be an essential part of the evaluation, as they may explain variations of effects across different contexts. They can help to describe *how* prevention interventions work and should be accurately identified and reported. Moreover, replications of evaluation studies in different contexts should be promoted and considered as an element of quality.
- Research-oriented. It should require an accurate reporting of underpinning theories, contents, mechanisms of action, and effects of single components on target behaviours to support the advancement of prevention science.
- Transparent and open access. All steps of evaluation should be transparently reported; descriptive information and complete data about evidence, benefits, risks, and variations related to different populations and contexts should be publicly available. The level of descriptive information must be sufficient to allow replications across different contexts with a high level of fidelity.
- Based on international cooperation. An international consensus on standards for releasing the certification of effectiveness is required to ensure widespread acceptance of this system in the scientific community. Therefore, a collaborative action of an extensive range of researchers, policy-makers, and health professionals is needed, as well as an extraordinary effort and mobilisation of resources.

**Figure 1:**
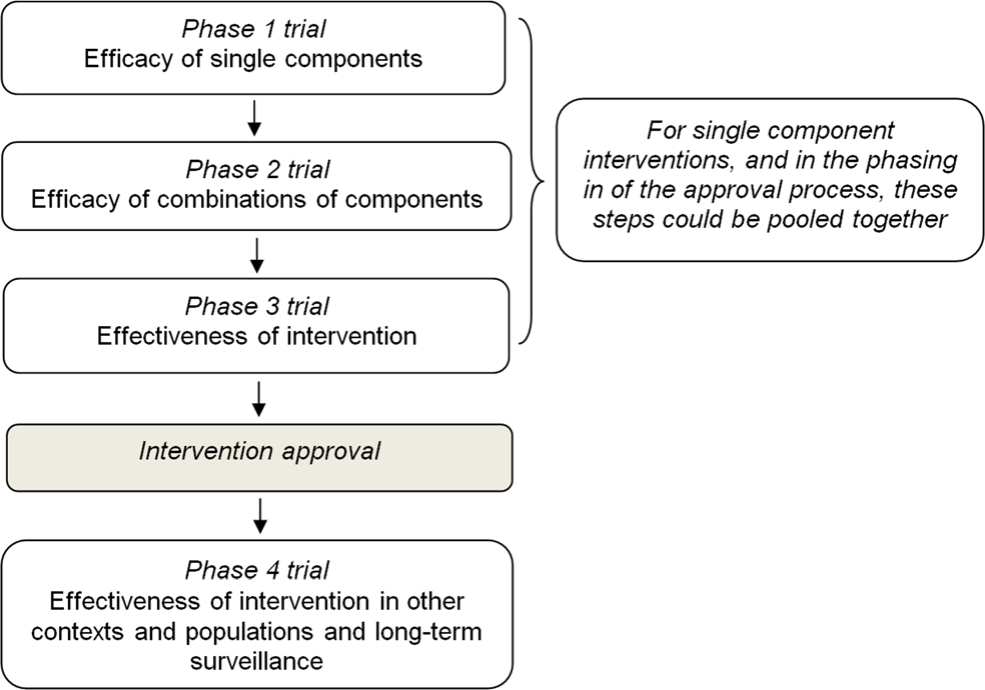
Proposal for a four-step evaluation and approval process of prevention interventions for health-compromising behaviours.

In light of existing experiences, and taking into account the key characteristics described above, a four-phase evaluation and approval process could be proposed (Figure 1):

- Phase 1 should be aimed at evaluating the effect of single components on mediators and short-term outcomes through experimental or observational studies. This phase should also assess dosage features (e.g., delivery frequency, duration, etc.) and other delivery characteristics such as the appropriate age group.
- Phase 2 should be aimed at evaluating the effect of combinations of single components which passed phase 1 on short-term outcomes in the target population through a pilot experimental study.
- Phase 3 should be aimed at evaluating the effectiveness of the whole intervention, once individual components have shown evidence of effectiveness on short- and medium-term outcomes in phase 2. Whenever possible, an adequately powered, randomized, controlled design should be used to allocate individuals or target groups (e.g., schools, families) to study arms. But, since environmental interventions can hardly be evaluated by a randomized study, and they constitute a cornerstone of any comprehensive prevention strategy, such as smoking bans or taxation of sugar-sweetened beverage, they should be assessed with other studies of high validity, as for example, cohort studies or interrupted time series.
- Interventions found to be effective in this phase should be approved for implementation and dissemination.
- Phase 4 should be aimed at evaluating the effectiveness of approved intervention in real-world settings (e.g., when delivered by a school team rather than a research team), the sustainability of effects on outcomes over a longer period of time and the long-term safety, and the replicability of effects on outcomes in different sociocultural contexts and populations, for which an adapted version of the intervention is usually needed.

Such a centralized system could be managed by a new public body, similar to the European Medicines Agency (EMA). Alternatively, an extended mandate to carry out such a process could be given to an existing and recognized public international agency or organization or to a network of research institutions coordinated by an international agency. The structural dimension of the proposed system cannot be easily estimated, but would be small. To make a rough estimation, an elaboration from the Cochrane Library can be of help: in 2012 the Library contained altogether 30 systematic reviews on primary prevention interventions; out of 503 interventions evaluated, only 171 (34.0%) showed at least one outcome favouring intervention [33]. Since Cochrane Library covers studies published in the last several decades and only studies showing positive results are expected to be submitted to such an approval process, these data suggest a few dozen interventions to be reviewed per year.

The funding requirements are a critical point: the amount depends too much on the ambition of the project, and cannot be estimated, even crudely. In any case, in analogy with a scientific journal, all the processes could be managed by a central editorial unit, supported by a network of referees, which would considerably contain costs.

Once an intervention has been approved, it should be included in a repository of effective interventions. The system would provide all needed materials and contacts with developers and trainers, together with the necessary information to select the intervention fitting the prevention needs (such as target behaviour, population, and setting), and contextual constraints (e.g., availability of human resources, time, and funding) of practitioners, decision-makers, and policymakers. The approval of a specific intervention can be nothing else than a strong recommendation to use the intervention. Nevertheless, with the progress of the project, and once the repository is populated sufficiently to be useful for all major conditions, we could expect that, at a country level, specific policies could be elaborated in order to promote the adoption of approved interventions.

## Conclusions

Prevention research has made considerable methodological advances in the past decades. This is not reflected in a parallel improvement of practice, largely due to a lack of regulatory systems for transferring evidence into practice.

A possible exception is the Framework Convention on Tobacco Control (www.who.int/fctc), with which WHO produced a strong frame of effective actions for tobacco control. However, such a convention still remains an exception and can be hardly expected to be reproduced for other risk behaviours. The need to address the overall deficit in rigorous evaluation of prevention interventions for health-compromising behaviours is thus pressing in all other fields of prevention in Europe and beyond.

This paper aims to initiate a debate about how best to develop a central, transparent, public, and evidence-based system of evaluation and approval of prevention interventions for health-compromising behaviours in Europe. A four-phase approval process is outlined and is intended to foster further discussion.

This approval process would result in a repository of effective prevention interventions to be recommended to European Union member states for adoption, in order to base prevention strategies on scientific evaluations. Policy-makers and people working in the prevention field would find in this repository interventions and programmes to address the prevention needs of the target populations, together with all documents and materials useful to apply them. Furthermore, the repository would be even more useful for non-European and developing countries having similar health problems, for which the building of any systematic evaluation system for prevention is not foreseen for obvious economic reasons.

To steer the evaluation activities of prevention interventions in a transparent approval system would be a great progress not only for prevention practice but also for prevention science: the approval system would encourage evaluation, without which an intervention would not be included; would contribute to the standardization of evaluation methods; and would make available to the scientific community all the reports of the assessments, including component evaluation and mediation analysis. This could have the power to strongly improve research and give a contribution towards progressive learning on how prevention works.

Finally, the supply of effective and efficient behavioural interventions to prevention practice and policy making, in Europe but also elsewhere, would likely be a cost-effective initiative with a large expected impact on population health.
